# Adaption of a trigger tool to identify harmful incidents, no harm incidents, and near misses in prehospital emergency care of children

**DOI:** 10.1186/s12873-024-01125-4

**Published:** 2024-11-13

**Authors:** Niclas Packendorff, Carl Magnusson, Christer Axelsson, Magnus Andersson Hagiwara

**Affiliations:** 1 Prehospen–Centre for Prehospital Research, Faculty of Caring Science, Work Life and Social Welfare, University of Borås, Borås, Sweden; 2 Department of Prehospital Emergency Care, Sahlgrenska University Hospital, Gothenburg, Sweden

**Keywords:** Emergency medical services, Ambulance, Patient safety, Trigger tool, Children, Harmful incidents, Adverse events

## Abstract

**Background:**

The emergency medical service (EMS) addresses all chief complaints across all ages in various contexts. Children in EMS present a particular challenge due to their unique anatomical and physical properties, which require specific training that EMS clinicians often report lacking. This combination exposes children to incidents threatening patient safety. The most common method to highlight incidents is the incident reporting system. Studies have shown underreporting of such incidents, highlighting the need for multiple methods to measure and enhance patient safety in EMS for children. Thus, the aim of this study was to modify and adapt the current Ambulance TT for road-based EMS (ATT) to a pediatric version (pATT) with a guide containing definitions of triggers.

**Methods:**

The adaption of the ambulance trigger tool to a version suitable for children followed a stepwise manner, including (1) a review of previous literature to pinpoint areas of risk regarding patient safety among children in EMS. (2) Three sessions of expert panel discussions via video meetings were held to evaluate each trigger of the ATT in terms of clinical relevance, comprehensibility, language and areas of risk regarding patient safety among children in EMS. (3) Clinical use of the pATT along with Retrospective Record Review (RRR). (4) Calculation of Item-level validity index and positive predictive value (PPV) for each trigger. (5) calculate inter-rater reliability between two independent record reviewers.

**Results:**

The literature search revealed 422 respective 561 articles in Cinahl and Medline where headlines and abstracts were read to identify areas posing risks to patient safety in EMS for children. During the structured discussions, one trigger was added to the existing 19 derived from the ATT, and the trigger definitions were modified to suit children. The three most common triggers identified in the 900 randomly selected records were deviation from treatment guidelines (63.9%), incomplete documentation (48.3%), and the patient is non conveyed after EMS assessment (41.1%). The positive triggers were categorized into near miss (54.6%), no harm incident (5.8%), and harmful incident (0.4%). Inter-rater reliability testing showed excellent agreement.

**Conclusion:**

This study demonstrates the adaptation of an existing trigger tool (ATT) to one suitable for children. It also shows that the trigger tool, along with retrospective record review, is a feasible method to evaluate patient safety in EMS, thus complementing existing methods.

**Supplementary Information:**

The online version contains supplementary material available at 10.1186/s12873-024-01125-4.

## Background

The Emergency medical services (EMS) encounter patients with all chief complaints across all ages and in complex environments. This includes children with various degrees of medical, surgical, and traumatic complaints. Children are particularly vulnerable during different stages of development due to their unique anatomical and physiological characteristics. The need to calculate drug dosages and use properly sized equipment, combined with a lack of language skills to express their needs and symptoms, makes the assessment and resuscitation of children in need of EMS challenging [1, 2]. Children represent a small percentage (5.4–13%) of the total number of EMS missions, and among those, 6.4% were considered life-threatening [3, 4]. It has been suggested that the average EMS clinician encounters a critically ill teen, child, or infant every 625 to 1,087 days, making it a rare event [5]. In addition to the low incidence of children in need of resuscitation, EMS clinicians also report a lack of training, stress, and low confidence in assessing and resuscitating a child [6–8]. The combination of low incidence and inadequate training exposes children in the EMS to increased risk for patient safety incidents.

Various methods to measure incidents and work for increased patient safety are available today, and one of the most common is the incident reporting system (IRS). The IRS is a voluntary and anonymous way to report incidents threatening patient safety within a healthcare organisation, but it has drawbacks, such as underreporting due to different barriers [9]. It has been suggested that an IRS only identifies 1–8% of the incidents in an intrahospital context [10–12]. The use of a single source of measurement may create false reassurance regarding patient safety within an organisation and is reactive in its form [10, 13]. Therefore, employing multiple methods is essential for identifying patients at risk, allowing for proactive measures to be taken to enhance patient safety. Thus, an IRS can be complemented with the use of a trigger tool (TT) together with Retrospective Record Review (RRR) to identify incidents where the Global Trigger Tool (GTT) is one of the most common [14].

The frequency and types of incidents regarding children in EMS have previously been evaluated with different methods. Record reviews in transports deemed as critical showed incidents in 70.1% of the reviewed records where 23.3% were potentially severe. The incidents were associated with clinical reasoning, airway management/resuscitation, medications, and equipment. The risk for severe incidents was higher if the child was below the age of one year or in cardiopulmonary arrest [2]. Another study investigated airway management in children by record review and showed that the use of bag-valve-mask ventilation, endotracheal intubation, and the use of airway adjuncts are areas of risk and associated with incidents, especially during cases with cardiopulmonary arrest [15]. Similar types of incidents were found during simulated resuscitation of the critically ill child. Evaluation of EMS clinician’s performance revealed a lack of airway/ventilation support, failure to use proper equipment size, and miscalculation of the child’s weight, resulting in both under and overdosages regarding medications [16–19].

The frequency of incidents in hospitals varies according to reported findings. An American study on hospitalized children showed a frequency of 1% regarding harmful incidents, of which 0.6% were considered preventable. The types of harmful incidents were birth and diagnostic-related [20] Another study in the pediatric emergency department (ED) showed that 2.5% suffered a harmful incident related to management/diagnostic issues and suboptimal follow-up [21].

To our knowledge, there is no Trigger Tool (TT) developed for a general EMS population of children. We have previously developed and published a TT for adults. Thus, the aim of this study is to [1] modify and adapt the current Ambulance TT (ATT) for road-based EMS [22] to a pediatric version (pATT) with a guide containing definitions of triggers [2]. use the pATT on randomly selected ambulance patient records to present frequencies regarding positive triggers and incidents [3]. Calculate the Item-level validity index and positive predictive value (PPV) for each trigger and [4] calculate inter-rater reliability between two independent record reviewers.

## Method

### Design

**Fig. 1:**
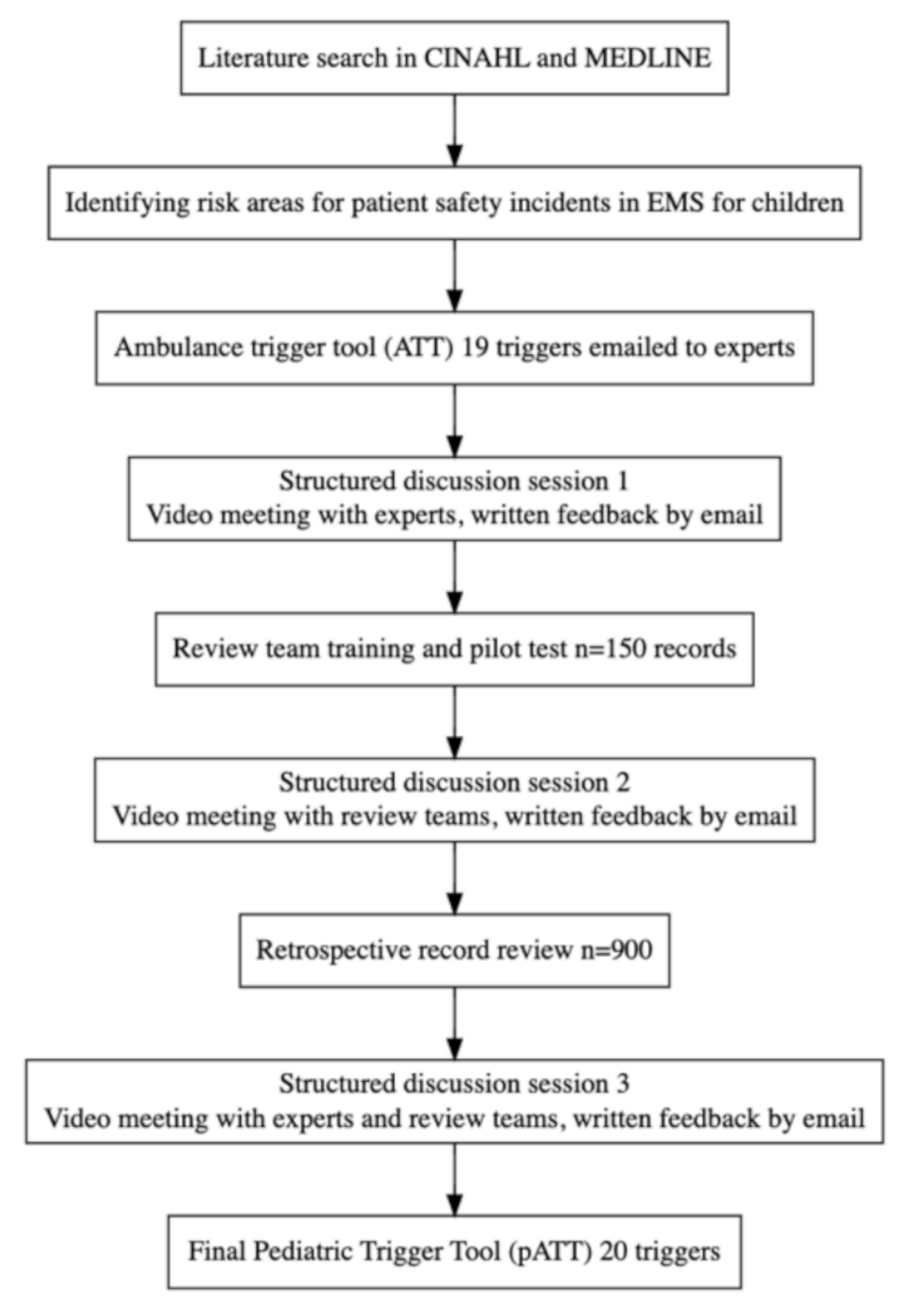
Flow chart of the development process of the pediatric EMS trigger tool. EMS, emergency medical services. RRR, retrospective record review

The development of the pATT followed a stepwise manner including (1) a review of previous literature to pinpoint areas of risk regarding patient safety among children in EMS. (2) Three sessions of expert panel discussions with video meetings. (3) Clinical use of the pATT along with Retrospective Record Review (RRR) (Fig. 1).

### Setting

The Swedish EMS service is funded by general taxes and organized by different regions, each of which may have multiple EMS organisations with varying clinical guidelines and documentation systems. At the national level, every ambulance must be staffed by a registered nurse (RN), who usually holds a master’s degree in ambulance, intensive, or anesthesiologic care.

During the development of the pATT, which lasted from January 3, 2023, to May 31, 2024, we included three EMS organisations that participated in the study by reviewing records of pediatric patients with the pATT. To obtain diversity in EMS allocation, mission length, and patient demographics, we included EMS organisations operating in urban, rural and a mixed organisation. EMS Gothenburg is located in an urban area with a population of 770,000, a median mission time of 70 min, and 85,000 EMS missions each year. EMS region Dalarna was situated in a rural area with a population of 281,000, a median mission time of 76 min, and 56,500 EMS missions each year. The EMS region Skåne represented a mix of rural and urban areas, with a population of 1.41 million, 204,000 EMS missions, and a median mission length of 66 min.

### Terminology and definitions

We utilized the term incident as defined by the World Health Organisation (WHO) [23], which is further divided into near miss (NM), No harmful incident (NHI) and harmful incident (HI). Additionally, the incidents were categorized as AB (could pose a risk of harm) to I (the incident contributed to the patient’s death) according to the National Coordinating Council for Medication Error Reporting and Prevention (NCC MERP) index [24].

A NM is an incident that did not reach the patient but could pose a risk of harm. For example, incomplete documentation, lack of vital sign registrations or clinical examination that were omitted but required according to clinical guidelines for the actual patient chief complaint. These incidents are categorized as A-B according to the NCC MERP.

A NHI is an incident that did reach the patient but did not cause any harm. For example, a child with symptoms of infection who is not conveyed but admitted to the ED within 72 h without septicemia but receives treatment with antibiotics/and/or observation then returns home. This categorizes as (C) ‘An incident that affected the patient but did not cause any harm’, or (D) ‘An incident that affected the patient and demanded observation or treatment to assure that no harm occurred’.

A HI also known as adverse event, is an incident that caused harm to the patient and is categorized as; (E) ‘contributed to or resulted in temporary harm and required intervention’; (F) ‘contributed to or resulted in temporary harm requiring outpatient care, readmission or prolonged hospital care’; (G) ‘contributed to or caused permanent patient harm’; (H) ‘An event that required lifesaving intervention within 60 min’; (I) ‘contributed to the patient’s death’. For example, a child with symptoms of infection who is not conveyed but admitted to the ED within 72 h with septicemia, which requires treatment at the hospital (intensive care or ordinary ward).

### Step 1. Establish areas threatening patient safety through a literature search

To develop the pATT version from the existing ATT, the areas threatening patient safety regarding children needed to be evaluated. A literature search was performed in CINAHL and MEDLINE to pinpoint these areas using keywords such as patient safety, prehospital, ambulance, trigger tool, adverse events, pediatric, children, and neonatal in different combinations (see supplement 1). Headlines and abstracts were reviewed in search results containing fewer than 100 articles. Literature describing threats to patient safety in children was included and read as a whole. No further validation of the literature was utilized. The literature was used to assess if the current ATT could detect such incidents in the upcoming structured discussions.

### Step 2. Expert panel and structured discussions

The expert panel consisted of seven experts divided between three registered nurses (RNs) and four medical doctors (MDs). The recruitment process was conducted via email, and the experts were considered eligible if they had experience in EMS and/or ED and patient safety. The registered nurses had previous experience working with patient safety TTs, as well as experience in EMS. The medical doctors had previous experience as children’s ED/intensive care unit/EMS physicians. They were also involved in work for increased patient safety within their organisations or at a national level. They were familiar with other methods like incident reporting and root cause analysis.

Structured discussions with a moderator were used to define and achieve consensus regarding the triggers and definitions of the pATT. The discussions were moderated by the first author, who was also responsible for inviting and ensuring the participation of all experts, with meetings scheduled based on polling. Expert and consensus methodologies are considered suitable for complex research problems, with the fundamental idea that the collective opinion of a group of experts is stronger than each individual opinion [25]. An expert is considered a professional within their field and has knowledge on a specific topic [26, 27].

The 19 triggers and definitions of the first ATT version were emailed to the seven experts two weeks before the first session. The experts were also provided with inspirational literature describing patient safety incidents in EMS [2, 5, 19], along with the current pediatric trigger tool from SKR [28].

The three sessions were conducted with a moderated face-to-face video meeting and a shared screen technique where each trigger was constantly viewable by the experts. The moderator asked the experts to evaluate each trigger definition according to clinical relevance, comprehensibility, language, and risk areas in the context of children in EMS. Each definition was changed instantly with red text and accepted by the experts before moving on to the next trigger definition. The session was video recorded for the ability to evaluate the expert opinions if needed. When completed, the pATT with its changes was sent by email to each expert for evaluation with the ability to provide written feedback. Written feedback was merged into one document and emailed once again until all experts approved the document. Consensus was reached when all experts had approved the pATT by email, and no further changes were made.

### Step 3. The retrospective record review (RRR) and review teams

The use of a TT requires access to the patient’s records once the care has been completed. An RN (primary reviewer) conducts a review of the record, searching for positive triggers. A positive trigger is a clue indicating a potential incident. If positive triggers are found, they are evaluated to determine if they resulted in an incident or not. Incidents with no risk of harm are categorized as near misses or no harmful incidents, and according to NCC MERP steps AB and C. Incidents with a risk of harm are presented to the MD for secondary review, and no categorization is done by the primary reviewer.

The MD reviews the record presented by the RN and determines whether the patient has been harmed. If the incident is considered a harmful incident, categorization is done according to the NCC MERP E to I, specifying the type of harm and assessing whether the harmful incident was preventable. If no harm is identified, the MD may decide that no incident has occurred or use the categorization AB, C, and D.

The three EMS organisations formed review teams consisting of a registered nurse (RN) and a medical doctor (MD). The RN’s had a master’s degree in ambulance care and were active in road-based EMS within their organisation. The MDs were medical directors of their organisation thus accustomed with investigating incidents reported by the IRS.

Within each organisation, the RNs randomized records meeting the inclusion criteria of (1) a primary mission where clinical reasoning takes place and (2) being ≤ 17 years of age during two different sessions: a pilot study and one final RRR. In the pilot study, the review teams randomized and reviewed 50 records per organisation for training purposes. These records were not included in the final analysis or result presentation. The teams were tasked with evaluating not only the methodology but also the clinical relevance, comprehensibility, and language of the triggers, which would serve as a foundation for the teams to gain experience in the RRR methodology and regarding the triggers and their definitions. These experiences were used in the structured discussions to discuss and adapt the triggers.

The final RRR consisted of 300 randomized and reviewed records per organisation, aligning with the Institute for Healthcare Improvement’s recommendation of reviewing at least 20 records per month [14]. The randomization was performed using the Mersenne Twister algorithm in Excel^®^ [29]. The aim was to use the pATT on randomized ambulance records to present frequencies regarding positive triggers and incidents, but also to further evaluate the triggers more comprehensively based on experiences used in the structured discussions. The pATT was created digitally as a database within Microsoft Access^®^ for the convenience of the review teams.

### Review team training

Each member of the review teams received the handbook describing the TT methodology developed by the Swedish Association of Local Authorities and Regions (SKR) [30], along with the pATT definitions. The material explains the common terminology used in the trigger tool context, such as positive triggers, incidents, and categorizations according to the NCC MERP. During online video led by the first author, the review teams received training in the trigger tool methodology where each trigger of the pATT preview was discussed regarding aim and possible scenarios for the trigger to become positive. The review teams had the opportunity to ask questions about the methodology and received training on how to randomize records and practice using the database in Microsoft Access^®^. The review teams also received training using the pATT in clinical practice during the pilot study of 150 records. Consultation was available by telephone and email by the author throughout the study.

### Analysis

The frequency of positive triggers, NM, NHI, and HI, is presented frequency-based according to the WHO and the NCC MERP. To calculate the item-level content validity index (I-CVI), the members of the review teams reviewed and evaluated each trigger according to clinical relevance, comprehensibility, and utility and answered a form with a 4-point Likert scale, where 1 = not relevant, 2 = somewhat relevant, 3 = quite relevant, and 4 = highly relevant. The I-CVI was calculated by summing the number of reviewers grading trigger 3 or 4 and dividing by the total number of reviewers. I-CVI of 0.80 or higher was considered highly relevant [31]. The positive predictive value (PPV) calculation was done by dividing the number of times a trigger resulted in a NM, NHI, and HI, by the total times the trigger was found and multiplying by 100 [32].

Two primary reviewers independently reviewed the same 90 records, randomized from the 300 records of one organization, to calculate inter-rater reliability, using the triggers as variables for the calculation. Positive and negative triggers were totalled in a confusion matrix for Cohen’s kappa analysis [33]. The nature of the pATT will cause an overrepresentation of negative triggers, causing a low Cohen’s kappa due to the prevalence problem [34]. Therefore, it is complemented with the prevalence-adjusted and bias-adjusted kappa (PABAK) [35]. Kappa values of 0.21–0.40, 0.41–0.60, 0.61–0.80, and 0.81–1.00 were respectively considered fair, moderate, substantial, and almost perfect [36]. The interpretation of PABAK and Cohen’s kappa are identical [37]. All analyses were performed with R studio version 2023.12.1 + 402.

## Results

### Literature review

The literature search revealed 422 and 561 articles in CINAHL and MEDLINE, respectively, where headlines and abstracts were read (see supplement 1). Headlines and abstracts revealing areas posing risk for patient safety was read as a whole. The articles identified several areas posing risks to patient safety in EMS for children. These include interventions during the resuscitation of critically ill children, such as airway management, ensuring proper sizing of equipment, accurate estimation of weight, appropriate dosages of drugs, and the use of restraints during transport (see supplement 1). These areas were evaluated by the experts and incorporated into the pATT in the following structured discussions.

### Structured discussion session one

The following adaptations to the 19 triggers were made to the pATT during session one:

B1A Assessment/Interventions according to SX-ABCDE (scene safety, eXanguinating bleeding, Airway, Breathing, Circulation, Disability, Exposure) were added with absence of inhalation treatment when the child is obstructive, and seizures left untreated.

B1B Assessment/Interventions for specific conditions were adapted, the non-administration of acetylsalicylic acid to a patient with acute coronary syndrome was removed.

B1C Absence of measured vital signs were added with capillary refill to complement blood pressure.

B1D Absence of relevant clinical examination was added, with a general impression evaluating the child’s appearance (awake, playful, decreased response to stimuli, loss of muscle tone), work of breathing (effortlessly, grunting, intercostal retractions, or abdominal muscle use), circulation to skin (normal colour, pallor, mottling, cyanosis). Weight specification regarding infants and when subjected to drug administration. Nutritional examination (suction reflex) and elimination (diuresis), when assessing infants.

B2 Physical injury during patient transport was renamed to B2 The child transported without restraints/physical harm during the transfer and added to be positive if the child was transported without restraints specific to children.

B3 Deterioration of patient’s condition during transport were revised with vital signs and cut-offs specific to children at different ages.

B4 Telephone interpreter has not been used in case of language deficiency were added with caregiver.

B5 Inconsistency between the EMS clinicians and emergency physician’s assessment and triage were adapted with the removal of a positive trigger when the patient is taken directly definitive treatment regarding neurology such as thrombolysis and thrombectomy or cardiology percutan coronar intervention.

B6 The patient is non conveyed after EMS assessment was added with the ability to not become positive if the patient returns within 72 h for the same chief complaint if it is a part of the treatment, for example observation at home instead of the ED.

L1 Unfavourable/Inappropriate drug treatment were added to become positive if wrong dosage were administered in relation to the child’s weight.

Structured discussion session two.

With the experience received from the pilot study of 150 records, the review teams discussed the pATT and made the following adaptations to the triggers during the session:

A1 Incomplete documentation was added with additional explanation under each criterion for a positive trigger. Information regarding the patient’s identity with personal identification number and type of ID verification (Relative, Passport, ID card). Essential details about the events preceding contact with EMS with the reason for EMS contact and the progression of the chief complaint. Information about the suspected condition and the reason for more significant interventions with clinical status according to ABCDE and clinical reasoning regarding chief complaint/working diagnosis/differential diagnosis. Essential details about interventions taken and planned actions with clinical reasoning motivating decisions regarding patient treatment and optimal level of care. Information about the information provided to the patient and the decisions made regarding treatment options and the possibility of a renewed medical assessment with self-care advice and information regarding symptoms and the time frame for when the patient should contact healthcare services again for a renewed assessment, especially in the case of a positive B6.

A3 Time on site > 10 min in life-threatening conditions was added to consider the patient’s condition in relation to time on site. For example, time on site > 10 min in a life-threatening condition where the condition does not require a longer time on site.

B1A Assessment/Interventions according to SX-ABCDE were altered by the removal of surgical interventions such as cricothyrotomy and thoracotomy/stomy for the trigger to be positive.

B1B Assessment/Interventions for specific conditions were added to complement B1A, becoming positive if fluid administration was needed without an established hypotension/shock covered by B1A.

B1C Absence of measured vital signs was added to become positive if no complementing vital signs were measured when the first set was deviant or if p-ketones were not measured when indicated by the chief complaint.

B1D Absence of relevant clinical examination was complemented with the consideration of the groins and testicles as a genesis for abdominal pain in the child.

B5 Inconsistency between the EMS clinicians and emergency physician’s assessment and triage were renamed to inconsistency between the EMS clinicians and the receiving physician’s assessment and triage.

B9 Report of concern was added to the list of triggers. The trigger is considered positive if a report of concern/contact with social services/police has not been carried out despite signs of physical/psychological violence, neglect, presence of alcohol/drugs, for example, intoxication in the guardian.

L1 Unfavorable/Inappropriate drug treatment was added to the definition become positive if there was no evaluation of the drug effect after treatment.

### RRR with 900 records

The demographics from the RRR of 900 records are shown in Table 1 divided by organisation. The median age of the children was 7, with a range of 2–14 (25th and 75th quantiles). 47% (*n* = 423) were female and 55.1% (*n* = 496) were dispatched as prio 1 (lights and sirens). Among all children assessed 59% (*n* = 530) were transported to hospital or primary care after assessment, the remainder were non conveyed. The most common mode of transport was by ambulance (85%, *n* = 451) and the majority (82%, *n* = 371) was not assessed as prio 1 by the EMS clinician. The most common chief complaint was trauma (22.3%, *n* = 201).

**Table 1:** Demographics of the RRR (*n* = 900)

	Rural organisation (*n* = 300)	Mixed organisation (*n* = 300)	Urban organisation (*n* = 300)
**Age Median (quantile)** ^**a**^	6.5 (2–14)	6 (1–14)	7 (1–14)
**Sex** ***n*** **(%)**			
Female	141 (47) ^c^	138 (46)	144 (48)
Male	158 (52.7)	162 (54)	156 (52)
**Dispatcher priority** ***n*** **(%)** ^b^			
Prio 1	129 (43)	206 (68.7)	161 (53,7)
Prio 2	150 (50)	92 (30.7)	134 (44.7)
Prio 3	21 (7)	2 (0.7)	5 (1.7)
**Initial assessment of level of care** ***n*** **(%)**			
Hospital or primary care	165 (55)	202 (67.3)	163 (54.3)
Stay on scene	135 (45)	98 (32.7)	137 (45.7)
**Mode of transport to hospital** ***n*** **(%)**			
Ambulance	148 (89.7)	159 (78.7)	144 (88.3)
Patient transport	0 (0.0)	0 (0)	1 (0.6)
Seated transport	2 (1.2)	1 (0.5)	2 (1.2)
Own transportation	15 (9.1)	42 (20.8)	16 (9.8)
**Ambulance priority to hospital** ***n*** **(%)** ^b^			
Prio 1	28 (18.9)	30 (18.5)	22 (15.3)
Prio 2	118 (79.7)	113 (71)	97 (67.4)
Prio 3	2 (1.4)	16 (10.5)	22 (15.3)
Prio 4	0 (0)	0 (0)	3 (2.1)
**Top five prehospital field assessment** ***n*** **(%)** ^**c**^			
Trauma	89 (29.7)	65 (21.7)	47 (15.7)
Seizures	32 (10.7)	32 (10.7)	47 (15.7)
Respiratory distress/dyspnoea/breathing difficulties	57 (19)	48 (16)	46 (15.3)
Allergy	10 (3.3)	6 (2)	31 (10.3)
Infection	29 (9.7)	34 (11.3)	29 (9.7)

^a^ 25th and 75th quantiles

^b^ Prio 1 (lights and sirens)

^c^ 1 missing

The frequency of positive triggers found in the RRR and the PPV grouped by incidents are presented in Table 2. The most common triggers were A1 Incomplete documentation 48.3% (*n* = 435), B1 Deviations from treatment guidelines 63.9% (*n* = 575) sub grouped by B1C Absence of measured vital signs 48.2% (*n* = 434), with the most common being multiple vital signs (23%) followed by blood pressure/capillary refill (17.8%). B1D was positive in 28.7% (*n* = 258) of the records, with the most common reason being the lack of specifying the child’s weight when administering medication. The trigger B6 Patient care is terminated after the ambulance nurse’s assessment showed that the children was non conveyed in 41.1% (*n* = 370). The categorization according to incidents and NCC MERP area shown in Table 3 where near miss (AB) was the most frequent occurring, 54.6% (*n* = 491). The I-CVI for each trigger are presented in Table 4.

**Table 2:** Positive triggers and positive predictive value (PPV) for each trigger grouped by near miss, no harmful incident and harmful incident after reviewing patient records in the three EMSa organisations (*n* = 900)

	Positive triggers detected in primary review *n* (%)	Positive trigger related to near miss *n*	PPV related to near miss (%)	Positive trigger related to no harmful incident *n*	PPV related to no harmful incident (%)	Positive trigger related to harmful incident *n*	PPV harmful incident (%)
A1 Incomplete documentation	435 (48.3)	277	63.7	21	4,8	2	0.5
A2 Response time > 20 min priority 1 (lights and sirens)	21 (2.3)	12	57.1	8	38.1	0	0
A3 Time on site > 10 min in case of life-threatening conditions	16 (1.8)	4	25	5	31.2	1	6.2
A4 Breakdown or faulty/missing equipment	2 (0.2)	1	50	1	50	0	0
A5 Shortage of EMS resources	2 (0.2)	1	50	0	0	1	50
A6 Other	2 (0.2)	0	0	1	0	0	0
B1 Deviations from treatment guidelines	575 (63.9)	414	72	40	7	3	0.5
B1A Assessment/Interventions according to SX-ABCDE^b^	8 (0.9)	0	0	3	37.5	3	37.5
B1B Assessment/Interventions in specific conditions	19 (2.1)	6	31.6	10	52.6	0	0
B1C Absence of measured vital signs	434 (48.2)	311	71.7	29	6,7	2	0.5
B1D Absence of relevant clinical examination	258 (28.7)	198	76.7	15	5.8	1	0.4
B2 The child transported without restraints/physical harm	0 (0.0)	0	0	0	0	0	0
B3 Deterioration of patient’s condition during transport	7 (0.8)	1	14.3	1	14.3	1	14.3
B4 Telephone interpreter has not been used in case of language deficiency	10 (1.1)	9	90	1	10	0	0
B5 Inconsistency between the EMS clinicians and the receiving physician’s assessment and triage	7 (0.8)	1	14.3	1	14.3	0	0
B6 The patient is non-conveyed after the EMS assessment	370 (41.1)	227	61.4	14	3.8	0	0
B6 Patient return within 72 h	42 (4.7)	23	54.8	11	26.2	0	0
B7 Alternative mode of transport to definitive care	80 (8.9)	29	48.8	4	5.0	1	1.2
B8 Ambulance destination deviates from local guidelines	5 (0.6)	3	60	1	20	0	0
B9 Report of concern	14 (1.6)	1	7.1	10	71.4	0	0
L1 Unfavourable/Inappropriate drug treatment	6 (0.7)	2	33.3	2	33.3	0	0

^a^ Emergency Medical Services

^b^ Scene safety, eXanguinating bleeding, Airway, Breathing, Circulation, Disability, Exposure

**Table 3:** Categorization of incidents after reviewing patient records in the three EMS a organisations (*n* = 900)

Incidents according to NCC MERP^b^ *n* (%)		Incidents according to WHO^c^ *n* (%)	
		No incident	353 (39.2)
AB	491 (54.6)	Near miss	491 (54.6)
C	52 (5.8)	No harm incident	52 (5.8)
D	0 (0.0)		
E	2 (0.2)	Harmful incident	4 (0.4)
F	1 (0.1)		
G	0 (0.0)		
H	1 (0.1)		
I	0 (0.0)		

^a^Emergency Medical Services

^b^National Coordination Council for Medication Error Reporting and Prevention index

^c^World Health Organisation

**Table 4:** Triggers with I-CVI a after reviewing patient records in the three EMS b organisations (*n* = 900)

	Clinical relevance (CVI)	Comprehensibility (CVI)	Utility (CVI)
A1 Incomplete documentation	1	1	1
A2 Response time > 20 min priority 1 (lights and sirens)	0.4	1	0.6
A3 Time on site > 10 min in case of life-threatening conditions	1	1	0.8
A4 Breakdown or faulty/missing equipment	1	1	0.8
A5 Shortage of EMS resources	0.8	0.8	0.6
A6 Other	1	1	1
B1 Deviations from treatment guidelines	0.8	1	1
B1A Assessment/Interventions according to SX-ABCDE^c^	1	1	0.8
B1B Assessment/Interventions in specific conditions	0.8	0.8	0.6
B1C Absence of measured vital signs	0.8	1	1
B1D Absence of relevant clinical examination	1	1	1
B2 The child transported without restraints/physical harm	0.6	0.8	0.6
B3 Deterioration of patient’s condition during transport	1	0.6	0.6
B4 Telephone interpreter has not been used in case of language deficiency	0.6	0.8	0.4
B5 Inconsistency between the EMS clinicians and the receiving physician’s assessment and triage	0.8	1	0.6
B6 The patient is non-conveyed after the EMS assessment	1	1	0.8
B7 Alternative mode of transport to definitive care	0.6	1	0.8
B8 Ambulance destination deviates from local guidelines	1	1	1
B9 Report of concern	1	1	0.8
L1 Unfavourable/Inappropriate drug treatment	1	1	1

^a^ Item-level Content Validity Index

^b^ Emergency Medical Services

^c^ Scene safety, eXanguinating bleeding, Airway, Breathing, Circulation, Disability, Exposure

### IRR session based on 90 records

The session of IRR testing between two primary reviewers presented substantial agreement (Table 5). Cohen’s kappa k = 0.78 and PAPAK k = 0.88.

**Table 5:** Inter-rater reliability between two reviewers’ triggers when reviewing identical patient records (*n* = 90)

		RN ^a^ 1	
		Positive	Negative
RN 2	Positive	232	33
	Negative	68	1467
	Cohen’s D 0.78	Accuracy 0.94	PABAK^a^ 0.88

^a^ Registered nurse

^b^ PABAK = Prevalence-adjusted and bias-adjusted kappa

### Structured discussion session three

With the experience received from the RRR of 900 records, the review teams along with the experts discussed the pATT and made the following adaptations to the triggers:

A2 Response time > 20 min priority 1 (lights and sirens) was added with prio 1 A and 1B to represent the current indexing in Sweden being prio 1 A (cardiac arrest), prio 1B (unconsciousness).

B1B Assessment/Interventions for specific conditions were added with misinterpretation of a time-sensitive condition on the ECG. Failure to administer pain relief if VAS (visual analogue scale) > 3 or an equivalent scale adapted for children. Failure to administer treatment according to the plan for advanced cardiac and pulmonary resuscitation.

B1D Absence of relevant clinical examination regarding chief complaint syncope was complemented with further explanation (loss of consciousness and/or vegetative symptoms) concerning the indication for an ECG. Infants was added with age specification 0–12 month.

B2 The child transported without restraints/physical harm was renamed to B2 Harm during transfer/transport.

B5 Inconsistency between the EMS clinicians and the receiving physician’s assessment and triage was renamed to B5 Inconsistency between the EMS clinicians and the receiving departments assessment and triage.

L1 Unfavorable/Inappropriate drug treatment was added to the definition to become positive if the child received > 1 drugs excluding over-the-counter drugs.

See supplement 2 for a translated version of the pATT.

## Discussion

The most common triggers found in the RRR were A1 Incomplete documentation, B1 Deviations from treatment guidelines, and B6 The patient is non conveyed after EMS assessment. Incomplete documentation rarely contributes to an HI but generates high frequencies of NMs thus indicating an area of improvement in the EMS documentation. We found incomplete documentation in 48% of the children’s records and it has been previous suggested that the EMS documentation in Sweden is incomplete in 14–60% [22, 38]. Incomplete documentation affects patient safety because it summarizes the interaction between the clinician and the patient and is of importance for patient handover from EMS to the ED to guide medical decisions [39, 40].

The B1 Deviation from treatment guidelines was positive in 63.9% of the records and is further divided into the triggers B1A, B1B, B1C, and B1D. Among these, B1C: Absence of measured vital signs was the most frequent, positive in 48.2% of the records. This indicates that vital signs were partly or totally missing in these records, with only 52.8% containing respiratory rate (RR), pulse oximetry (PO), blood pressure/capillary refill (BP), heart rate (HR), and temperature. One study found that RR, PO, BP, and HR were recorded in 80% of EMS records across all ages and 70% among infants [41]. Another study found a complete set of vital signs in 21% of infants, 51% of children aged 1–5 years, 73% of children aged 6–11 years, and 75% of children aged 12–17 years, indicating that measuring vital signs is problematic among young children [42]. The lack of on scene vital signs has been associated with increased mortality in the patient presenting with traumatic injury [43]. Triage systems are also dependent on documented vital signs where studies have shown incomplete triage in the paediatric patient [44, 45]. B6 The patient is non-conveyed after EMS assessment triggered in 41.1% of the records and previous studies have reported between 14 and 30.3% [45, 46]. No harmful incident was associated with the trigger and the high frequency of non-conveyed children of the sample also highlights the rare event of a child in need of resuscitation.

The pATT showed high frequencies of near misses (54.6%) in the records, mainly originating from incomplete documentation and deviation from treatment guidelines, indicating areas of improvement in the EMS. No harmful incidents that reached the patient but did not cause harm were found in 5.8% of the records, and harmful incidents were found in 0.4%. The incidence of harmful incidents in EMS for children in a randomized sample of chief complaints remains unclear, and future studies are needed. Intrahospital studies suggest that 1-4.87% of hospitalized children suffers a harmful incident [20, 47].

This study evaluated the performance of each trigger categorized by incident. Although the positive predictive value (PPV) for each trigger can be considered low in relation to HIs, we believe this is mainly due to the randomized sample of all chief complaints, where children rarely present with a critical condition requiring resuscitation. The trigger B1A: Assessment/Interventions according to SX-ABCDE showed higher PPV values than the other triggers in relation to HIs. It represents interventions required for a child in need of resuscitation, such as airway management and ventilatory support—areas known to be insufficient. Previous studies have evaluated trigger performance using PPV in a pediatric hospital setting, mainly focusing on detecting HIs, finding a variety ranging from 0 to 100% [48, 49]. Thus, the PPV is highly dependent on the frequency of HIs in the sample studied.

This study describes the adaptation of the current TT for EMS to a version suitable for children. The methodology involved a review of existing literature, structured discussions with an expert panel, and a RRR. These structured discussions were used to examine, adapt, and reach consensus on the triggers, emphasizing the collective opinion of the group over individual perspectives. Several consensus methodologies using experts exist, with one of the most well-known being the Delphi technique [25]. The traditional Delphi method typically involves experts completing quantifiable surveys anonymously in each round, with the surveys being iteratively modified until consensus is reached [50, 51]. However, various modifications to the traditional Delphi approach have led to confusion in the methodology, and a narrow perspective of the method may inhibit valuable aspects [52]. Given the complexity and length of the trigger definitions, we opted for face-to-face discussions to reach consensus, deviating from the traditional Delphi methodology where elements such as anonymity are considered key to mitigating issues related to hierarchy and group dynamics, which may prevent experts from expressing their opinions [52, 53]. While this approach could be viewed as a limitation, it can also be advantageous, allowing for the immediate addressing of complex problems and details during trigger discussions. Additionally, it may reduce the issues related to declining response rates often observed with questionnaires [26, 54]. We believe that some of the anonymity-related issues were mitigated by moderating each session, encouraging each member to voice their opinion, and allowing the experts to provide written feedback on the pATT after each session. Furthermore, the trigger definitions were shared individually with the experts before the meeting, enabling them to reflect on their opinions. The face-to-face discussions were far more fruitful and versatile than the written feedback in this study. The use of face-to-face meetings has been successfully implemented in previous studies and in the development of TTs [32, 55, 56]. It is also used in various methodologies, such as the nominal group technique and focus groups [57, 58].

The discussions were conducted via video conferencing, enabling experts from geographically dispersed locations to participate in this study. Without video conferencing, progressing with this study would have been considerably more challenging. The optimal size of an expert panel has been debated in the literature, with no consensus reached [52, 59]. This study utilized a panel of seven experts, supported by six reviewers. We believe that this panel size, along with the reviewers, was sufficient to facilitate rich and versatile discussions regarding the triggers. During the study period, there was limited experience in using a TT along with RRR in Sweden. As a result, inviting panel members with limited knowledge posed a risk of trigger removal with the potential for identifying harm. Nevertheless, we believe that the discussed triggers cover most aspects of EMS, thereby guiding the reviewers in identifying incidents.

## Strength and limitations

This study demonstrates the adaptation of an existing TT to one suitable for children and applies retrospective record review RRR on a randomized sample of children in EMS. To our knowledge, this is the first study with a randomized sample of children in need of EMS who do not have a critical chief complaint.

There are several limitations when conducting studies using the RRR method, where the data relies on records written by EMS clinicians. Several triggers, such as B1A: Assessment/Measures according to SX-ABCDE, B2: Harm during transfer/transport, and L1: Unfavorable/Inappropriate drug treatment, are difficult to evaluate due to the current design of EMS records in Sweden. The routine practice to specify the weight of the child during resuscitation in records does not exist, making it problematic to evaluate equipment size or drug dosages accurately. The pATT is designed with a patient perspective and identifies areas of risk in the EMS environment, without considering the current documentation culture. Therefore, it provides EMS organisations with frequencies and the ability to address these limitations.

## Conclusion

Children in the context of EMS pose a risk of incidents threatening patient safety, which a reporting system can fail to recognize. This study offers a TT adapted for children in road-based EMS, providing a feasible method to complement other approaches for evaluating patient safety in EMS. The TT can be used alongside the RRR for a general sample, as well as for different samples with specific aims, such as non-conveyed children in EMS. The TT can be the subject of future research, including the refinement of triggers, further validation, and mapping of the frequency of triggers and incidents in children. This can complement the incident reporting system and support the continuous evaluation of patient safety.

## Electronic supplementary material

Below is the link to the electronic supplementary material.

Supplementary Material 1

Supplementary Material 2

## Data Availability

The datasets generated and analyzed during the current study are not publicly available but are available from the corresponding author on reasonable request.
