# Supplementary material for: Adaption of a trigger tool to identify harmful incidents, no harm incidents, and near misses in prehospital emergency care of children
Source: BMC Emerg Med. 2024 Nov 13;24:213. doi: 10.1186/s12873-024-01125-4 (PMC11559164; doi:10.1186/s12873-024-01125-4)
Supplement: Supplementary file 1 — Supplementary Material 1 [file 12873_2024_1125_MOESM1_ESM.pdf]

Supplement 1 Includes articles and literature search

Abebe, Y., Hetmann, F., Sumera, K., Holland, M., & Staff, T. (2020). The effectiveness and safety of paediatric prehospital pain management: a systematic review. J Trauma Resusc Emerg Med, 29, 170. https://doi.org/10.1186/s13049-021-00974-3

Bahr, N., Meckler, G., Hansen, M., & Guise, J. M. (2021). Evaluating pediatric advanced life support in emergency medical services with a performance and safety scoring tool. The American Journal of Emergency Medicine, 48, 301–306. https://doi.org/10.1016/J.AJEM.2021.06.061

Bernius, M., Thibodeau, B., Jones, A., Clothier, B., & Witting, M. (2008). Prevention of pediatric drug calculation errors by prehospital care providers. Prehospital Emergency Care, 12(4), 486–494. https://doi.org/10.1080/10903120802290752

Curtis, K., Kennedy, B., Holland, A. J. A., Tall, G., Smith, H., Soundappan, S. S. V., Burns, B., Mitchell, R. J., Wilson, K., Loudfoot, A., Dinh, M., Lyons, T., Gillen, T., & Dickinson, S. (2019). Identifying areas for improvement in paediatric trauma care in NSW Australia using a clinical, system and human factors peer-review tool. Injury, 50(5), 1089–1096. https://doi.org/10.1016/j.injury.2019.01.028

Ciarletta, J., Lillvis, D., Stoklosa, A., Kasper, B., & Bass, K. (2024). Safe Ground Transport of Pediatric Patients: A Qualitative Assessment of Best Practice Guidelines Implementation. Prehospital Emergency Care, 28(2), 282–290. https://doi.org/10.1080/10903127.2023.2227249

Cottrell, E. K., O'Brien, K., Curry, M., Meckler, G. D., Engle, P. P., Jui, J., Summers, C., Lambert, W., & Guise, J.-M. (2014). Understanding Safety in Prehospital Emergency Medical Services for Children. Prehospital Emergency Care, 18(3), 350–358. https://doi.org/10.3109/10903127.2013.869640

Cushman, J. T., Fairbanks, R. J., O'Gara, K. G., Crittenden, C. N., Pennington, E. C., Wilson, M. A., Chin, N. P., & Shah, M. N. (2010). Ambulance personnel perceptions of near misses and adverse events in pediatric patients. Prehospital Emergency Care, 14(4), 477–484. https://doi.org/10.3109/10903127.2010.497901

Duby, R., Hansen, M., Meckler, G., Skarica, B., Lambert, W., & Guise, J.-M. (2018). Safety Events in High Risk Prehospital Neonatal Calls. Prehospital Emergency Care, 22(1), 34–40. https://doi.org/10.1080/10903127.2017.1347222

Fidacaro, G. A., Jones, C. W., & Drago, L. A. (2020). Pediatric Transport Practices Among Prehospital Providers. Pediatric Emergency Care, 36(11), e632–e635. https://doi.org/10.1097/PEC.0000000000001564

Hansen, M., Meckler, G., Lambert, W., Dickinson, C., Dickinson, K., van Otterloo, J., & Guise, J.-M. (2016). Patient safety events in out-of-hospital paediatric airway management: a

medical record review by the CSI-EMS. BMJ Open, 6(11), e012259. https://doi.org/10.1136/bmjopen-2016-012259

Hansen, M., Meckler, G., O'Brien, K., Engle, P., Dickinson, C., Dickinson, K., Jui, J., Lambert, W., Cottrell, E., & Guise, J.-M. (2016). Pediatric Airway Management and Prehospital Patient Safety. Pediatric Emergency Care, 32(9), 603–607. https://doi.org/10.1097/PEC.0000000000000742

Hansen, M., Meckler, G., Lambert, W., O'Brien, K., Dickinson, C., Dickinson, K., van Otterloo, J., & Guise, J. M. (2018). Out-of-Hospital Pediatric Patient Safety Events: Results of the CSI Chart Review. Prehospital Emergency Care, 22(3), 290–299. https://doi.org/10.1080/10903127.2017.1371261

Hansen, M., Meckler, G., Lambert, W., Dickinson, C., Dickinson, K., van Otterloo, J., & Guise, J.-M. (2016). Patient safety events in out-of-hospital paediatric airway management: a medical record review by the CSI-EMS. BMJ Open, 6(11), e012259. https://doi.org/10.1136/bmjopen-2016-012259

Hansen, M., Meckler, G., O'Brien, K., Engle, P., Dickinson, C., Dickinson, K., Jui, J., Lambert, W., Cottrell, E., & Guise, J.-M. (2016). Pediatric Airway Management and Prehospital Patient Safety. Pediatric Emergency Care, 32(9), 603–607. https://doi.org/10.1097/PEC.0000000000000742

Herzberg, S., Hansen, M., Schoonover, A., Skarica, B., Mcnulty, J., Harrod, T., Snowden, J. M., Lambert, W., & Guise, J. M. (2019). Association between measured teamwork and medical errors: an observational study of prehospital care in the USA. BMJ Open, 9(10). https://doi.org/10.1136/bmjopen-2018-025314

Hoyle, J. D., Crowe, R. P., Bentley, M. A., Beltran, G., & Fales, W. (2017). Pediatric Prehospital Medication Dosing Errors: A National Survey of Paramedics. Prehospital Emergency Care, 21(2), 185–191. https://doi.org/10.1080/10903127.2016.1227001

Hoyle, J. D., Davis, A. T., Putman, K. K., Trytko, J. A., & Fales, W. D. (2012). Medication dosing errors in pediatric patients treated by emergency medical services. Prehospital Emergency Care, 16(1), 59–66. https://doi.org/10.3109/10903127.2011.614043

Hoyle, J. D., Ekblad, G., Hover, T., Woodwyk, A., Brandt, R., Fales, B., & Lammers, R. L. (2020). Dosing Errors Made by Paramedics During Pediatric Patient Simulations After Implementation of a State-Wide Pediatric Drug Dosing Reference. Prehospital Emergency Care, 24(2), 204–213. https://doi.org/10.1080/10903127.2019.1619002

Hoyle, J. D., Sleight, D., Henry, R., Chassee, T., Fales, B., & Mavis, B. (2016). Pediatric Prehospital Medication Dosing Errors: A Mixed-Methods Study. Prehospital Emergency Care, 20(1), 117–124. https://doi.org/10.3109/10903127.2015.1061625

Hubble, M. W., Brown, L., Wilfong, D. A., Hertelendy, A., Benner, R. W., & Richards, M. E. (2010). A Meta-Analysis of Prehospital Airway Control Techniques Part I: Orotracheal and Nasotracheal Intubation Success Rates. Prehospital Emergency Care, 14(3), 377–401. https://doi.org/10.3109/10903121003790173

King, B. R., & Woodward, G. A. (2002). Pediatric critical care transport--the safety of the journey: a five-year review of vehicular collisions involving pediatric and neonatal transport teams. Prehospital Emergency Care, 6(4), 449–454. https://doi.org/10.1080/10903120290938111

Lammers, R., Byrwa, M., & Fales, W. (2012). Root causes of errors in a simulated prehospital pediatric emergency. Academic Emergency Medicine, 19(1), 37–47. https://doi.org/10.1111/j.1553-2712.2011.01252.x

Meckler, G., Hansen, M., Lambert, W., O'Brien, K., Dickinson, C., Dickinson, K., van Otterloo, J., & Guise, J. M. (2018). Out-of-Hospital Pediatric Patient Safety Events: Results of the CSI Chart Review. Prehospital Emergency Care, 22(3), 290–299. https://doi.org/10.1080/10903127.2017.1371261

Meckler, G., Leonard, J., & Hoyle, J. (2014). Pediatric Patient Safety in Emergency Medical Services. Clinical Pediatric Emergency Medicine, 15(1), 18–27. https://doi.org/10.1016/j.cpem.2014.01.003

Nehme, E., Nehme, Z., Cox, S., & Smith, K. (2022). Outcomes of paediatric patients who are not transported to hospital by Emergency Medical Services: a data linkage study. Emergency Medicine Journal, 40(1), 12–19. https://doi.org/10.1136/emermed-2022-212350

Schoonover, A., Eriksson, C. O., Nguyen, T., Meckler, G., Hansen, M., Harrod, T., & Guise, J. M. (2022). A chart review tool to systematically assess the safety of prehospital care for children with out-of-hospital cardiac arrest. Journal of the American College of Emergency Physicians Open, 3(3). https://doi.org/10.1002/emp2.12726

Stoklosa, A. R., Zafron, M. L., Bass, K. D., & Lillvis, D. (2023). Safe Pediatric Ground Ambulance Transport. International Journal of Paramedicine, 2, 19–28. https://doi.org/10.56068/mesg1218

Tiyyagura, G. K., Gawel, M., Alphonso, A., Koziel, J., Bilodeau, K., & Bechtel, K. (2017). Barriers and Facilitators to Recognition and Reporting of Child Abuse by Prehospital Providers. Prehospital Emergency Care, 21(1), 46–53. https://doi.org/10.1080/10903127.2016.1204038

Walker, D., Moloney, C., SueSee, B., Sharples, R., Blackman, R., Long, D., & Hou, X.-Y. (2023). Factors Influencing Medication Errors in the Prehospital Paramedic Environment: A Mixed Method Systematic Review. Prehospital Emergency Care, 27(5), 669–686. https://doi.org/10.1080/10903127.2022.2068089

Wang, H. E., Lave, J. R., Sirio, C. A., & Yealy, D. M. (2006). Paramedic Intubation Errors: Isolated Events Or Symptoms Of Larger Problems? Health Affairs, 25(2), 501–509. https://doi.org/10.1377/hlthaff.25.2.501

Wells, M., Henry, B., & Goldstein, L. (2023). Weight Estimation for Drug Dose Calculations in the Prehospital Setting – A Systematic Review. Prehospital and Disaster Medicine, 38(4), 471–484. https://doi.org/10.1017/S1049023X23006027

MY EBSCOhost

Wednesday, August 16, 2023 3:06:06 PM

#	Query	Limiters/Expanders	Last Run Via	Results
S26	S3 AND S5 AND S8	Search modes - Find all my search terms	Interface - EBSCOhost Research Databases<br>Search Screen - Advanced Search<br>Database - CINAHL	6
S25	S3 AND S5 AND S7	Search modes - Find all my search terms	Interface - EBSCOhost Research Databases<br>Search Screen - Advanced Search<br>Database - CINAHL	25
S24	S3 AND S5 AND S6	Search modes - Find all my search terms	Interface - EBSCOhost Research Databases<br>Search Screen - Advanced Search<br>Database - CINAHL	17
S23	S3 AND S4 AND S8	Search modes - Find all my search terms	Interface - EBSCOhost Research Databases<br>Search Screen - Advanced Search<br>Database - CINAHL	0
S22	S3 AND S4 AND S7	Search modes - Find all my search terms	Interface - EBSCOhost Research Databases<br>Search Screen - Advanced Search<br>Database - CINAHL	0
S21	S3 AND S4 AND S6	Search modes - Find all my search terms	Interface - EBSCOhost Research Databases<br>Search Screen - Advanced Search<br>Database - CINAHL	0
S20	S2 AND S5 AND S8	Search modes - Find all my search terms	Interface - EBSCOhost Research Databases<br>Search Screen - Advanced Search<br>Database - CINAHL	2

S19	S2 AND S5 AND S7	Search modes - Find all my search terms	Interface - EBSCOhost Research Databases<br>Search Screen - Advanced Search<br>Database - CINAHL	36
S18	S2 AND S5 AND S6	Search modes - Find all my search terms	Interface - EBSCOhost Research Databases<br>Search Screen - Advanced Search<br>Database - CINAHL	22
S17	S2 AND S4 AND S8	Search modes - Find all my search terms	Interface - EBSCOhost Research Databases<br>Search Screen - Advanced Search<br>Database - CINAHL	0
S16	S2 AND S4 AND S7	Search modes - Find all my search terms	Interface - EBSCOhost Research Databases<br>Search Screen - Advanced Search<br>Database - CINAHL	1
S15	S2 AND S4 AND S6	Search modes - Find all my search terms	Interface - EBSCOhost Research Databases<br>Search Screen - Advanced Search<br>Database - CINAHL	1
S14	S1 AND S4 AND S8	Search modes - Find all my search terms	Interface - EBSCOhost Research Databases<br>Search Screen - Advanced Search<br>Database - CINAHL	10
S13	S1 AND S4 AND S7	Search modes - Find all my search terms	Interface - EBSCOhost Research Databases<br>Search Screen - Advanced Search<br>Database - CINAHL	36
S12	S1 AND S4 AND S6	Search modes - Find all my search terms	Interface - EBSCOhost Research Databases<br>Search Screen - Advanced Search<br>Database - CINAHL	38
S11	S1 AND S2 AND S8	Search modes - Find all my search terms	Interface - EBSCOhost Research Databases	7

			Search Screen - Advanced Search<br>Database - CINAHL	
S10	S1 AND S2 AND S7	Search modes - Find all my search terms	Interface - EBSCOhost Research Databases<br>Search Screen - Advanced Search<br>Database - CINAHL	142
S9	S1 AND S2 AND S6	Search modes - Find all my search terms	Interface - EBSCOhost Research Databases<br>Search Screen - Advanced Search<br>Database - CINAHL	79
S8	neonatal	Search modes - Find all my search terms	Interface - EBSCOhost Research Databases<br>Search Screen - Advanced Search<br>Database - CINAHL	81,078
S7	children	Search modes - Find all my search terms	Interface - EBSCOhost Research Databases<br>Search Screen - Advanced Search<br>Database - CINAHL	826,403
S6	pediatric	Search modes - Find all my search terms	Interface - EBSCOhost Research Databases<br>Search Screen - Advanced Search<br>Database - CINAHL	220,176
S5	adverse events	Search modes - Find all my search terms	Interface - EBSCOhost Research Databases<br>Search Screen - Advanced Search<br>Database - CINAHL	124,300
S4	trigger tool	Search modes - Find all my search terms	Interface - EBSCOhost Research Databases<br>Search Screen - Advanced Search<br>Database - CINAHL	2,093
S3	ambulance	Search modes - Find all my search terms	Interface - EBSCOhost Research Databases<br>Search Screen - Advanced	9,902

			Search<br>Database - CINAHL	
S2	prehospital	Search modes - Find all my search terms	Interface - EBSCOhost Research Databases<br>Search Screen - Advanced Search<br>Database - CINAHL	19,362
S1	patient safety	Search modes - Find all my search terms	Interface - EBSCOhost Research Databases<br>Search Screen - Advanced Search<br>Database - CINAHL	190,792

Saturday, August 26, 2023 8:00:49 AM

#	Query	Limiters/Expanders	Last Run Via	Results
S26	S3 AND S5 AND S8	Search modes - Find all my search terms	Interface - EBSCOhost Research Databases<br>Search Screen - Advanced Search<br>Database - MEDLINE	12
S25	S3 AND S5 AND S7	Search modes - Find all my search terms	Interface - EBSCOhost Research Databases<br>Search Screen - Advanced Search<br>Database - MEDLINE	35
S24	S3 AND S5 AND S6	Search modes - Find all my search terms	Interface - EBSCOhost Research Databases<br>Search Screen - Advanced Search<br>Database - MEDLINE	37
S23	S3 AND S4 AND S8	Search modes - Find all my search terms	Interface - EBSCOhost Research Databases<br>Search Screen - Advanced Search<br>Database - MEDLINE	0
S22	S3 AND S4 AND S7	Search modes - Find all my search terms	Interface - EBSCOhost Research Databases<br>Search Screen - Advanced Search<br>Database - MEDLINE	0
S21	S3 AND S4 AND S6	Search modes - Find all my search terms	Interface - EBSCOhost Research Databases<br>Search Screen - Advanced Search<br>Database - MEDLINE	0
S20	S2 AND S5 AND S8	Search modes - Find all my search terms	Interface - EBSCOhost Research Databases<br>Search Screen - Advanced Search<br>Database - MEDLINE	3

S19	S2 AND S5 AND S7	Search modes - Find all my search terms	Interface - EBSCOhost Research Databases<br>Search Screen - Advanced Search<br>Database - MEDLINE	43
S18	S2 AND S5 AND S6	Search modes - Find all my search terms	Interface - EBSCOhost Research Databases<br>Search Screen - Advanced Search<br>Database - MEDLINE	38
S17	S2 AND S4 AND S8	Search modes - Find all my search terms	Interface - EBSCOhost Research Databases<br>Search Screen - Advanced Search<br>Database - MEDLINE	1
S16	S2 AND S4 AND S7	Search modes - Find all my search terms	Interface - EBSCOhost Research Databases<br>Search Screen - Advanced Search<br>Database - MEDLINE	1
S15	S2 AND S4 AND S6	Search modes - Find all my search terms	Interface - EBSCOhost Research Databases<br>Search Screen - Advanced Search<br>Database - MEDLINE	2
S14	S1 AND S4 AND S8	Search modes - Find all my search terms	Interface - EBSCOhost Research Databases<br>Search Screen - Advanced Search<br>Database - MEDLINE	16
S13	S1 AND S4 AND S7	Search modes - Find all my search terms	Interface - EBSCOhost Research Databases<br>Search Screen - Advanced Search<br>Database - MEDLINE	66
S12	S1 AND S4 AND S6	Search modes - Find all my search terms	Interface - EBSCOhost Research Databases<br>Search Screen - Advanced Search<br>Database - MEDLINE	70
S11	S1 AND S2 AND S8	Search modes - Find all my search terms	Interface - EBSCOhost Research Databases	4

			Search Screen - Advanced Search<br>Database - MEDLINE	
S10	S1 AND S2 AND S7	Search modes - Find all my search terms	Interface - EBSCOhost Research Databases<br>Search Screen - Advanced Search<br>Database - MEDLINE	117
S9	S1 AND S2 AND S6	Search modes - Find all my search terms	Interface - EBSCOhost Research Databases<br>Search Screen - Advanced Search<br>Database - MEDLINE	116
S8	neonatal	Search modes - Find all my search terms	Interface - EBSCOhost Research Databases<br>Search Screen - Advanced Search<br>Database - MEDLINE	296,139
S7	children	Search modes - Find all my search terms	Interface - EBSCOhost Research Databases<br>Search Screen - Advanced Search<br>Database - MEDLINE	1,723,379
S6	pediatric	Search modes - Find all my search terms	Interface - EBSCOhost Research Databases<br>Search Screen - Advanced Search<br>Database - MEDLINE	1,282,914
S5	adverse events	Search modes - Find all my search terms	Interface - EBSCOhost Research Databases<br>Search Screen - Advanced Search<br>Database - MEDLINE	338,307
S4	trigger tool	Search modes - Find all my search terms	Interface - EBSCOhost Research Databases<br>Search Screen - Advanced Search<br>Database - MEDLINE	6,593
S3	ambulance	Search modes - Find all my search terms	Interface - EBSCOhost Research Databases<br>Search Screen - Advanced	20,750

			Search<br>Database - MEDLINE	
S2	prehospital	Search modes - Find all my search terms	Interface - EBSCOhost Research Databases<br>Search Screen - Advanced Search<br>Database - MEDLINE	20,963
S1	patient safety	Search modes - Find all my search terms	Interface - EBSCOhost Research Databases<br>Search Screen - Advanced Search<br>Database - MEDLINE	444,129
