# Supplementary material for: Adaption of a trigger tool to identify harmful incidents, no harm incidents, and near misses in prehospital emergency care of children
Source: BMC Emerg Med. 2024 Nov 13;24:213. doi: 10.1186/s12873-024-01125-4 (PMC11559164; doi:10.1186/s12873-024-01125-4)
Supplement: Supplementary file 2 — Supplementary Material 2 [file 12873_2024_1125_MOESM2_ESM.pdf]

Retrospective record review with a trigger tool

TRIGGERS AND DEFINITIONS EMS EDITION ADAPTED FOR CHILDREN

Disclaimer: This edition has been translated from Swedish by the author and not in any professional manner.

Content

Definitions 2
General Triggers 3
A1 Incomplete documentation 3
A2 Response Time >20 minutes for priority 1 (lights and sirens) 4
A3 Time on site >10 minutes in case of life-threatening conditions 4
A4 Breakdown or faulty/missing equipment 5
A5 Shortage of EMS resources 5
A6 Other 6
Assessment/Intervention Triggers 6
B1 Deviations from treatment guidelines 6
B1A Assessment/Interventions according to SX-ABCDE 7
B1B Assessment/Interventions for specific conditions 9
B1C Absence of measured vital signs 9
B1D Absence of relevant clinical examination 10
B2 Physical harm during patient transport 11
B3 Deterioration of patient's condition during transport 12
B4 Telephone interpreter has not been used in case of language deficiency 13
B5 Inconsistency between the EMS clinicians and the receiving departments assessment and triage 14
B6 The patient is non-conveyed after the EMS assessment 15
B7 Alternative mode of transport to definitive care 16
B8 Ambulance destination deviates from local guidelines 17
B9 Report of concern 18
Trigger related to drug administration 19
L1 Unfavorable/Inappropriate drug treatment 19

Definitions

Positive trigger

An indication that an incident has occurred and needs evaluation by the reviewer.

Incident

An event is considered deviant and categorized into a near miss, no harmful incident, and harmful incident according to the WHO Conceptual Framework for the International Classification for Patient Safety.

Near miss (NM)

According to the NCC MERP, an NM incident (e.g., lack of documentation) neither affects nor harms the patient but poses the risk of an error and is categorized as (A) or (B).

No harmful incident (NHI)

An NHI reaches the patient but does not cause harm (e.g., omission of electrocardiogram (ECG) in a patient with chest discomfort not diagnosed with acute coronary syndrome) affects the patient but does not cause harm. It is categorized as (C) 'An incident that affected the patient but did not cause any harm' or (D) 'An incident that affected the patient and demanded observation or treatment to assure that no harm occurred.'

Harmful incident (HI, adverse event)

A HI is an incident that harms the patient (adverse event). For example, the omission of an ECG in a patient with chest discomfort who is later diagnosed with ST-elevated myocardial infarction, thus delaying the time to definitive treatment. It is categorized as (E) 'Contributed to or resulted in temporary harm and required intervention'; (F) 'Contributed to or resulted in temporary harm requiring outpatient care, readmission, or prolonged hospital care'; (G) 'Contributed to or caused permanent patient harm'; (H) 'An event that required lifesaving intervention within 60 min'; or (I) 'Contributed to the patient's death.'

The process of RRR with a trigger tool

The primary reviewer reviews the record for positive triggers. If positive triggers are found, the primary reviewer assesses whether the positive trigger contributed to an incident and if it affected the patient. Incidents that did not affect the patient or pose any risk of harm are classified according to steps AB and C (Incidents Table 1). An incident with a risk of harm to the patient undergoes a secondary review by a physician. The primary reviewer does not classify such incidents in Table 1, leaving it to the secondary reviewer. The secondary reviewer evaluates incidents with a risk of harm and determines whether the patient has been harmed. If no harm occurred, the scale steps no incident, AB, C, and D are used (Table 1). If harm occurred, the type of harm (Table 2), the severity according to scale steps E to I (Table 3), and whether the harm was preventable (Table 4) are documented.

General Triggers

A1 Incomplete documentation	
**Definition**	**Incomplete documentation.**
**Considerations**	EMS clinicians are often the first healthcare providers to encounter the patient. The assessment can be crucial for the patient's ongoing contact with healthcare.<br><br>The trigger is considered positive if any of the following are missing:<br><br>• Information regarding the patient's identity<br>o Personal identification number.<br>o Type of ID verification (Relative, Passport, ID card).<br>• Essential details about the events preceding contact with EMS. Example:<br>o Reason for EMS contact and the progression of the chief complaint.<br>• Information about the suspected condition and the reason for more significant interventions. Example:<br>o Clinical status according to ABCDE.<br>o Clinical reasoning regarding chief complaint/working diagnosis/differential diagnosis.<br>• Essential details about interventions taken and planned actions. Example:<br>o Clinical reasoning motivating decisions regarding patient treatment and optimal level of care.<br>• Information about the information provided to the patient and the decisions made regarding treatment options and the possibility of a renewed medical assessment. Example:<br>o Self-care advice.<br>o Information regarding symptoms and the time frame for when the patient should contact healthcare services again for a renewed assessment, especially in the case of a positive B6.
**Incident**	A positive trigger is always considered an incident.
**Harm**	Incomplete documentation does not have to cause harmful incidents but is an important measure of quality.
**Preventability**	Incomplete documentation is considered an incident that is always preventable.

A2 Response Time >20 minutes for priority 1 (lights and sirens)	
**Definition**	**Prio 1 >20 minutes or other time agreed locally, for example rural areas.**
**Considerations**	The trigger is considered positive if:<br><br>• The response time to the patient exceeds 20 minutes, calculated from ambulance alert to arrival at the address, regardless of the cause.
**Incident**	A positive trigger is always considered an incident.
**Harmful incident**	Time-sensitive conditions that are delayed.
**Preventability**	An incident may be considered preventable if:<br>• The nearest available resource is already beyond >20 minutes at dispatch.

A3 Time on site >10 minutes in case of life-threatening conditions	
**Definition**	**Time on site >10 minutes in case of life-threatening conditions where the condition does not require a longer time at site.**
**Considerations**	When the patient presents with a life-threatening condition, the EMS clinicians have limited resources for definitive treatment. Initiating transport is crucial for conditions or injuries that can only be addressed at the hospital.<br><br>The trigger is considered positive if:<br><br>• Time on site >10 minutes from arriving at the patient to loading for a priority one to definitive care.
**Incident**	A positive trigger is always considered an incident.
**Harmful incident**	Delay to definitive treatment.
**Preventability**	Incidents are considered preventable if:<br><br>• The conditions were in place to transport the patient within 10 minutes, but there was no apparent reason for delay.

A4 Breakdown or faulty/missing equipment	
**Definition**	**Ambulance breakdown or faulty/missing technical or medical equipment that affects the patient's assessment or treatment.**
**Considerations**	The trigger is considered positive if:<br><br>• Breakdown of the ambulance that requires an additional ambulance to manage the patient.<br>• Faulty/missing technical or medical equipment, such as ECG, not readable or cannot be sent to a higher level of care, lack of map support.<br>• Vehicle collision.
**Incident**	A positive trigger is always considered an incident.
**Harmful incident**	It may not necessarily imply harm to the patient. Still, substandard assessment or treatment due to incorrect or missing equipment is a quality deficiency, and other types of harm may result.
**Preventability**	Incidents shall be considered preventable if:<br>• Ambulance maintenance not performed according to schedule.<br>• Incorrect or missing equipment that could have been detected during ambulance functional checks, such as an empty oxygen cylinder, forgotten emergency bag, or missing/expired medications.

A5 Shortage of EMS resources	
**Definition**	**Patient(s) demand for treatment exceeds available resources.**
**Considerations**	The trigger is considered positive if:<br><br>• EMS clinicians request additional resources that are not acquired due to a shortage of EMS resources.<br>• Due to a lack of resources, the patient receives a lower level of treatment than what is specified in the guidelines.
**Incident**	A positive trigger is always considered an incident.
**Harmful incident**	Patient(s) do not receive treatment according to guidelines due to shortage of resources.
**Preventability**	Incidents/harm are always considered preventable.

A6 Other	
**Definition**	**Any incident that is not covered by another trigger.**
**Considerations**	Incidents that are not identified by any other trigger are noted.<br>Describe the incident.

Assessment/Intervention Triggers

B1 Deviations from treatment guidelines	
**Definition**	**Deviations from treatment guidelines.**
**Considerations**	The EMS clinician uses an examination methodology according to SX-ABCDE and different clinical examination in relation to chief complaint, as well as various tools to assess and treat the patient presenting with various conditions. The purpose is to identify serious conditions where supportive treatment can be provided.<br><br>The trigger is considered positive if:<br><br>• The reviewer has reason to believe that deviations from treatment guidelines have been made. Further classification is made according to B2A, B2B or A6.
**Harmful incident**	Conditions that the reviewer perceives as serious such as acute coronary syndrome, stroke, sepsis, spinal cord injuries. Incorrect level of care that delays definitive treatment with risk of harm.
**Preventability**	Incidents shall be considered preventable if:<br>• There is no motivation for deviations from treatment guidelines.

B1A Assessment/Interventions according to SX-ABCDE	
**Definition**	**Deviation from treatment guidelines involving assessment and interventions according to SX-ABCDE.**
**Considerations**	EMS clinicians utilize an examination methodology according to SX-ABCDE to identify and address immediate life threats.<br><br>The trigger is considered positive if:<br><br>• Scene safety (S). Example: Lack of consideration for the scene where the patient is cared for.<br>• Catastrophic (X, arterial). Example: Failure to stop bleeding with pressure dressing/ tourniquet (TQ), wrong indication/application of TQ, or if the reviewer perceives that the management has not been performed correctly.<br>• Airway obstruction (A). Example: Failure to establish a clear airway using simple maneuvers or basic/advanced airway adjuncts, or if the reviewer perceives that the management has not been performed correctly.<br>• Ventilation issues (B). Example: Oxygen is not provided to a hypoxic patient, inhalation treatment is not given despite indication, apnea/hypoventilation/hypoxia despite therapy with oxygen not addressed with assisted ventilation/CPAP, or if the reviewer perceives that the management has not been performed correctly.<br>• Circulatory issues (C). For example, shock of various origins not addressed, or treatment not initiated, incorrect placement of mechanical compressions, intra-arterial or subcutaneous placement of an IV catheter, incorrect placement of an intraosseous needle, or if the reviewer perceives that the management has not been performed correctly.<br>• Neurological issues (D). Example: Decreased consciousness with a reversible cause, for example, hypoxia, hypoglycemia, intoxication not addressed, or if the reviewer perceives that the management has not been performed correctly.<br>• Exposure issues (E). Example: Severely hypothermic patient where warming has not been initiated, or if the reviewer perceives that the management has not been performed correctly.<br><br>Note that SX-ABCDE does not necessarily need to be written out in the record but can be assessed by

	the reviewer. For example, if history and vital parameters are present, the algorithm can be assessed as under control.
**Incident**	A positive trigger is always considered an incident.
**Harmful incident**	Omitted or incorrect interventions according to LX-ABCDE, airway injuries, pneumothorax/hemothorax, damage to internal organs, and thrombophlebitis.
**Preventability**	Incidents shall be considered preventable if:<br>• Care in the hazardous zone without adequate intervention.<br>• Catastrophic bleeding without adequate intervention.<br>• Respiratory problems without adequate intervention.<br>• Ventilation problems without adequate intervention.<br>• Circulatory problems without adequate intervention.<br>• Neurological problems (loss of consciousness) without adequate intervention.<br>• Exposure (protection against the hazard at the scene) without adequate intervention.<br>• Improper interventions due to the EMS clinician's lack of competence: Have additional resources been requested, or has a higher medical competence been consulted?

B1B Assessment/Interventions for specific conditions	
**Definition**	**Deviations from treatment guidelines for specific conditions that do not fall under interventions according to SX-ABCDE.**
**Considerations**	The trigger is considered positive if, for example:<br>• Absence of spinal movement restriction/stabilization when indicated.<br>• ECG shows a time-sensitive condition that is misinterpreted.<br>• Glucocorticoid not given in suspected Addison crisis.<br>• Failure to provide pain relief when VAS>3 or an equivalent scale adapted for children.<br>• A fluid bolus is not given without established shock when a documented need exists, such as a capillary refill greater than 4 seconds or when the chief complaint and clinical status reveal a fluid deficit.<br>• Omission of treatment according to the advanced cardiac life support plan, such as omitted medications despite indication.<br>• Or if the reviewer identifies other deviations.
**Incident**	A positive trigger is always considered an incident.
**Preventability**	Incidents shall be considered preventable:<br>• Improper interpretation of ECG.<br>• Absence of spinal movement restriction/stabilization when indicated or applied improperly. For example, the SRB significantly delays the time spent on the scene in the event of life-threatening injuries.<br>• Improper interventions due to lack of competence of the EMS clinician, have additional resources been requested or higher medical competence consulted?

B1C Absence of measured vital signs	
**Definition**	**Respiratory rate, saturation, blood pressure, pulse, level of consciousness and temperature are missing from documentation.**
**Considerations**	EMS largely relies on assessing and treating patients based on their vital signs.<br><br>The trigger is considered positive if:<br>• All or part of the patient's vital signs are missing<br>• Follow-up of abnormal vital signs is missing. Necessary for evaluation of trigger B3<br>P-glucose and ketones are included here if the chief complaint indicates it, such as decreased consciousness, seizures, or diabetes.
**Incident**	A positive trigger is always considered an incident.

**Harmful incident**	Absence of vital signs when the next healthcare provider detects abnormal vital signs that require treatment.
**Preventability**	Incidents shall be considered preventable:<br>• Vital signs have not been measured, and no relevant motivation has been provided for the omission.

**B1D Absence of relevant clinical examination**	
**Definition**	**Absence of clinical examinations relevant to the patient's chief complaint.**
**Considerations**	EMS clinicians rely on clinical examinations of the patient for their assessment and treatment.<br><br>Trigger are considered positive if:<br><br>• As detailed below, relevant clinical examinations are missing based on the chief complaint. Note that a nonspecific chief complaint may necessitate multiple examinations.
**Incident**	A positive trigger is always considered an incident.
**Harmful incident**	Conditions that could be detected by clinical examination, such as stroke, myocardial infarction, arrhythmias, and surgical diseases of the abdomen.
**Preventability**	Incidents shall be considered preventable if relevant clinical examinations have not been performed, such as:<br>• General impression (first view of the patient)<br>o Appearance (Awake, playful, decreased response to stimuli, loss of muscle tone).<br>o Work of breathing (Effortlessly, grunting, retractions, or abdominal muscle use).<br>o Circulation to skin (Normal color, cyanosis, paleness/pallor).<br>• ECG is not performed for the chief complaint of chest pain or syncope (when indicated, for example, loss of consciousness and/or vegetative symptoms).<br>• Pulmonary auscultation is not performed for the chief complaint of dyspnea.<br>• A neurological examination using an accepted scale is performed for chief complaint neurological symptoms.<br>• Abdominal examination (auscultation, palpation of the abdomen and/or testicles and groin) is not performed for chief complaint abdominal pain.

	• Nutritional examination (suction reflex) and elimination (diuresis) is not performed for infants (0–12 months)<br>• Weight specification is not performed for infants (0–12 months) and children of all ages undergoing drug administration.

**B2 Physical harm during patient transport**	
**Definition**	**Physical harm during patient transport.**
**Considerations**	EMS encounters patients with an elevated risk of falling. This risk can be due to various reasons, such as impairment of sensory function and chronic or acute illness.<br>The trigger is considered positive if:<br>• Harm occurs during transport, such as a fall, dropped stretcher
**Incident**	A positive trigger is always considered an incident.
**Harmful incident**	Fracture, bleeding, laceration, soft tissue or joint pain, concussion.
**Preventability**	Incidents shall be considered preventable if:<br>• The patient falls in the presence of the EMS clinicians.<br>• The lifting aid for patient transfer is not being used or used incorrectly.<br>• Restraints adapted for safe transports are not being used or are being used incorrectly.

**B3 Deterioration of patient's condition during transport**	
**Definition**	Deterioration of the patient's vital signs from previously measured values.<br>The trigger is considered positive if at least one of the following is met:<br>**Respiration**<br>• Respiratory arrest<br>• Saturation <90% with oxygen<br>• Respiratory rate<br>o <25 or >55 (0–3 months)<br>o <20 or >49 (4–11 months)<br>o <18 or >39 (1–2 years)<br>o <18 or >28 (3–5 years)<br>o <15 or >25 (6–11 years)<br>o <12 or >23 (12–15 years)<br>o <12 or >25 (16–18 years)<br>**Circulation**<br>• Cardiac arrest<br>• Systolic blood pressure <90 mm Hg<br>• Pulse<br>o <105 or >165 (0–3 months)<br>o <90 or >155 (4–11 months)<br>o <85 or >145 (1–2 years)<br>o <80 or >130 (3–5 years)<br>o <65 or >120 (6–11 years)<br>o <55 or >110 (12–15 years)<br>o <51 or >100 (16–18 years)<br>**Neurology**<br>• Glasgow/pediatric Coma Scale: drop >2 from baseline<br>• RLS >3<br>• Deterioration, according to AVPU<br>• P-glucose <3 mmol/L
**Considerations**	Investigate the events preceding the deterioration of the patient's condition, such as medication ingestion, self-mobilization, or a fall. Consider whether there is reason to believe that the deterioration was influenced by the EMS clinician's treatment or lack of treatment. Requires two sets of vital signs.
**Incident**	A positive trigger is always considered an incident. Should be secondary reviewed.
**Harmful incident**	Effects on respiration and circulation regardless of condition. For example, COPD/Asthma, Infection/sepsis, myocardial infarction, heart failure/pulmonary edema, trauma/bleeding.
**Preventability**	Incidents shall be considered preventable if:

	• The patient has not been monitored according to treatment guidelines or in accordance with their medical condition.<br>• Vital signs have not been adequately recognized, resulting in treatment being neglected or not initiated in a reasonable time.

**B4 Telephone interpreter has not been used in case of language deficiency**	
**Definition**	**Due to a language barrier, the EMS clinicians cannot communicate with the patient/caregiver, so they use a relative to gather the necessary information.**
**Considerations**	Patient history from the patient or caregiver is one of the cornerstones in the assessment and continued management of the patient in EMS. Interpretation with a relative can exaggerate and minimize the current chief complaint and history affecting the patient's management.<br><br>The trigger is considered positive if:<br><br>• Language deficiencies existed, and a telephone interpreter was not used. Key factors in the medical history are missing or distorted.
**Incident**	A positive trigger is always considered an incident.
**Harmful incident**	Any harmful incident that can be traced back to an inadequate medical history.
**Preventability**	Using a patient relative as an interpreter shall be considered preventable if:<br>• The situation allows contact with a telephone interpreter.

**B5 Inconsistency between the EMS clinicians and the receiving departments assessment and triage**	
**Definition**	**For time-sensitive/serious conditions, EMS clinicians and the receiving physicians do not agree on assessment and triage, resulting in delayed definitive care.**
**Considerations**	Emergency care aims to identify serious and time-sensitive conditions in the patient. EMS has limited capabilities for advanced diagnostics, and patient prioritization (triage) is based on medical history, vital signs, symptoms, and a few clinical examinations.<br><br>The trigger is considered positive if:<br><br>• The patient's triage color deviates from the emergency physician's assessment in the hospital, delaying the time to definitive treatment. For example, the patient may be given green or yellow priority but is then taken directly from the emergency department to definitive treatment such as emergency surgery or intensive care.<br><br>The purpose is to assess whether under-triage has occurred in the EMS.
**Incident**	A positive trigger is always considered an incident.
**Harmful incident**	Serious conditions not identified by EMS result in the patient being assigned a lower priority compared to the emergency physician's assessment.
**Preventability**	A low triage related to the patient's condition shall be considered preventable if:<br>• The EMS clinician has made a reasonable assessment, but the system for triage did not triage the patient correctly.<br>• The EMS clinician has assessed a lower triage than the triage system suggests.

B6 The patient is non-conveyed after the EMS assessment	
**Definition**	**The patient is assigned an ambulance resource, which results in the termination of care based on the EMS clinician's assessment.**
**Considerations**	Patients who remain at home face a risk of unmonitored deterioration or the possibility that a time-sensitive condition may not have been identified in the EMS clinician's assessment.<br><br>The trigger is considered positive if:<br><br>• The patient is non-conveyed.<br><br>Note the factors behind the decision, whether the contact was terminated by the EMS clinicians or the patient/guardian, if the patient/guardian was competent to make decisions (i.e., able to understand the consequences of the decision), and whether there was further planning for follow-up, such as with primary care. Additionally, note if the patient sought emergency medical care within 72 hours for the same symptoms and if any treatment was administered.
**Incident**	A positive trigger can be an incident if the patient seeks emergency medical care (ambulance, emergency department) within 72 hours for the same symptoms. The severity of the incident depends on the treatment administered.<br><br>Note that a new contact with EMS or the ED, in case of deterioration or lack of improvement, can be part of the planned treatment and does not necessarily indicate an incident by default. However, if there is reason to believe the care was terminated prematurely, it may warrant classification as an incident/harm of varying severity.
**Harmful incident**	Any condition where time to treatment is of importance.
**Preventability**	The incidents is considered preventable if:<br><br>• It appears that the EMS may have encouraged the patient to remain at home or seek a level of care where necessary interventions are not available.<br>• It appeared that the patient required emergency care but was non-conveyed.

B7 Alternative mode of transport to definitive care	
**Definition**	**The patient is transported to the emergency department (ED) by an alternative mode of transport instead of an ambulance after EMS assessment.**
**Considerations**	The trigger is considered positive if:<br><br>• The patient is transported to the ED by an alternative mode of transport. This does not necessarily involve an incident, but there is a risk of unattended deterioration and lack of treatment during transport.
**Incident**	A positive trigger is an incident if the patient's condition deteriorates during the alternative mode of transport.
**Harmful incident**	Harm resulting from the lack of treatment for failure of vital functions and undetected time-sensitive conditions, which leads to delays in definitive treatment.
**Preventability**	Incidents that occur in connection with an alternative mode of transport are considered preventable if:<br><br>• The chief complaint and/or patient status warranted ambulance transport, but an alternative mode of transport was selected.

B8 Ambulance destination deviates from local guidelines	
**Definition**	**The choice of hospital extends the patient's time to definitive treatment. Definitive treatment refers to procedures such as neurosurgery for a severe head injury, trauma surgery, or other specialist competencies regarding children.**
**Considerations**	The availability of specialist competencies varies depending on the region and population density. In urban areas, specialist competencies are available but are often concentrated in specific hospitals. Therefore, it is crucial for the patient to be transported directly to a hospital that can provide definitive treatment. There are situations where the nearest hospital should be chosen, such as an obstructed airway. Still, the general rule is that hospitals capable of providing definitive treatment should be prioritized. This may not be possible in rural areas requiring the reviewer's organizational knowledge.<br><br>The trigger is considered positive if:<br><br>• Despite an apparent need for specialist competence, where local guidelines prescribe transport to a specific hospital, the patient is transported to a hospital lacking this competence without a motivation for the decision.<br>• Secondary transport from the initial hospital to a higher level of care.
**Incident**	A positive trigger is always considered an incident. The severity depends on how the patient has been affected.
**Harmful incident**	Extended time to definitive treatment such as head injuries and high-energy trauma with multiple injuries.
**Preventability**	The incident is considered preventable if:<br><br>• The patient is transported to a hospital without specialist competence despite an apparent need, and no motivation supports the decision.

B9 Report of concern	
**Definition**	**Children who are being harmed or are at risk of harm because of a negative environment.**
**Consideration**	The trigger is considered positive if:<br><br>• A report of concern with social services/police is not made despite signs of physical/psychological abuse, neglect, or the presence of alcohol/drugs, such as intoxicated parents/guardians.
**Incident**	A positive trigger is always considered an incident.
**Harmful incident**	Any harm that can be attributed to the child's negative environment.
**Preventability**	The incident is considered preventable if:<br><br>• There are obvious signs that a child is in a negative environment, but no report of concern or/contact with social services/police is made, and no motivation is provided.

Trigger related to drug administration

L1 Unfavorable/Inappropriate drug treatment	
**Definition**	**Signs of unfavorable effects of drug treatment, such as hypersensitivity/anaphylaxis, antidote administration, inappropriate drug/dose, or incorrect method of drug administration.**
**Considerations**	Drug treatment can have negative effects on organs and vital functions, which may require the treatment to be unexpectedly discontinued or necessitate additional treatment to counteract these effects. Specific antidotes used to reverse the negative effects include naloxone for opioid overdose and flumazenil for benzodiazepine overdose.<br><br>Always review the administered drug dose in relation to weight.<br><br>The trigger is considered positive if:<br>• >1 drug administration (non-prescription).<br>• Lack of evaluation of the administered treatment.<br>• Incorrect dosage in relation to the child's weight.<br>• Interventions regarding ABCD following drug administration.<br>• The use of antidote following drug administration.<br>• Allergy/anaphylaxis following drug administration.<br>• The patient is administered the wrong drug<br>• If the reviewer experiences inappropriate drug treatment/dosage.
**Incident**	A positive trigger is always considered an incident.
**Harmful incident**	Allergies/anaphylaxis, confusion, loss of consciousness, respiratory distress, respiratory arrest, circulatory failure, and death.
**Preventability**	Incidents is considered preventable if:<br>• Medication is administered despite contraindications or known allergy/hypersensitivity.<br>• The risk of negative effects from interacting drugs has not been considered.<br>• Treatment with opioids or benzodiazepines has caused symptoms requiring antidote administration (e.g., naloxone, flumazenil).<br>• A transfusion reaction caused by errors during preparation or in connection with the transfusion.

Step-by-step user guide for retrospective record review with a trigger tool

1) **Identify records for review:**
   - Select records for review, either through random sampling or a selected sample.
2) **Use a review form for each record:**
   - Utilize a review form for each record, assigning a unique sequential number from a designated series that you create.
3) **Document patient ID and unique number:**
   - Document the patient's ID and the specific sequential number on a list maintained by the review team under journal confidentiality. This list facilitates the identification of a patient record if further analysis is required.
4) **Search for positive triggers in selected records:**
   - Examine the records for positive triggers. Refer to the definitions in the document on triggers and definitions. Mark the positive trigger in the review form with a "+" sign and note where the trigger was found in the record, along with the reason for the positive trigger.
5) **Primary reviewer notation of triggers and incidents:**
   - The primary reviewer reviews the record for positive triggers. If positive triggers are found, the primary reviewer assesses whether the positive trigger contributed to an incident and if it affected the patient. Incidents that did not affect the patient or pose any risk of harm are classified according to steps AB and C (Incidents Table 1). An incident with a risk of harm to the patient undergoes a secondary review by a physician. The primary reviewer does not classify such incidents in Table 1, leaving it to the secondary reviewer.
6) **Secondary reviewer assessment of incidents with a risk of harm:**
   - The secondary reviewer evaluates incidents with a risk of harm and determines whether the patient has been harmed. If no harm occurred, the scale steps no incident, AB, C, and D are used (Table 1). If harm occurred, the type of harm (Table 2), the severity according to scale steps E to I (Table 3), and whether the harm was preventable (Table 4) are documented.
7) **Documentation and summary of the record review:**
   - The RRR is documented in the review form and summarized, forming the basis for analyzing and planning for risk-reducing measures to enhance patient safety.

Assignment to work with retrospective record review with a trigger tool

---

EMS organization

Name personal identity number.

As of date, I have ______ been commissioned on behalf of the ambulance service to carry out a retrospective record review with a trigger tool as part of the work for increased patient safety. This work includes taking part in information on the various medical records and documentation systems the ambulance service has access to. The methodology for retrospective record review means that medical records from the ambulance service are subject to review in cases where the care occasion is also directly related to care at other clinics. The time the above person requires to complete their assignment is calculated to ______ hours per month. The assignment is valid until the end of the year. ______

Head of Operations (signature and clarification) *(date)*

EMS chief physician/equivalent (signature and clarification)

(date)

The assignment is drawn up in three identically signed copies, where the primary reviewer, the head of Operations, and the EMS Chief Physician/equivalent each keep one copy.

Review form, retrospective record review with trigger tool for EMS Children

Serial number: __________ Case number ___________

Time for EMS mission: _________

<table>
<tr><th></th><th>General Triggers</th><th>#</th><th colspan="5">Reason for positive trigger/ where in the record was the trigger found?</th></tr>
<tr><td>A1</td><td>Incomplete documentation</td><td></td><td colspan="5"></td></tr>
<tr><td>A2</td><td>Response time >20 minutes for priority 1</td><td></td><td colspan="5"></td></tr>
<tr><td>A3</td><td>Time on site >10 minutes in case of life-threatening conditions</td><td></td><td colspan="5"></td></tr>
<tr><td>A4</td><td>Breakdown or faulty/missing equipment</td><td></td><td colspan="5"></td></tr>
<tr><td>A5</td><td>Shortage of EMS resources</td><td></td><td colspan="5"></td></tr>
<tr><td>A6</td><td>Other</td><td></td><td colspan="5"></td></tr>
<tr><td></td><td>Assessment/Intervention Triggers</td><td></td><td colspan="5"></td></tr>
<tr><td>B1</td><td>Deviations from treatment guidelines</td><td></td><td colspan="5"></td></tr>
<tr><td>B1A</td><td>Assessment/Interventions according to SX-ABCDE</td><td></td><td colspan="5"></td></tr>
<tr><td>B1B</td><td>Assessment/Interventions for specific conditions</td><td></td><td colspan="5"></td></tr>
<tr><td>B1C</td><td>Absence of measured vital signs</td><td></td><td colspan="5"></td></tr>
<tr><td>B1D</td><td>Absence of relevant clinical examination</td><td></td><td colspan="5"></td></tr>
<tr><td>B2</td><td>Physical harm during patient transport</td><td></td><td colspan="5"></td></tr>
<tr><td>B3</td><td>Deterioration of patient´s condition during transport</td><td></td><td colspan="5"></td></tr>
<tr><td>B4</td><td>Telephone interpreter has not been used in case of language deficiency</td><td></td><td colspan="5"></td></tr>
<tr><td>B5</td><td>Inconsistency between the EMS clinicians and the receiving departments assessment and triage</td><td></td><td colspan="5"></td></tr>
<tr><td>B6</td><td>The patient is non-conveyed after EMS assessment</td><td></td><td>Cause</td><td>72H</td><td>Intervention</td><td>Hospitalized</td><td>Discharged</td></tr>
<tr><td>B7</td><td>Alternative mode of transport to definitive care</td><td></td><td colspan="5"></td></tr>
<tr><td>B8</td><td>EMS destination deviates from local guidelines</td><td></td><td colspan="5"></td></tr>
</table>

Continued. Review form, retrospective record review with trigger tool for EMS Children

Serial number: ___________ Case number: ____________

Time for EMS mission: _________

	Trigger related to drug administration		
L1	Unfavorable/Inappropriate drug treatment		

Number of incidents<br>Primary reviewer	______	Classification of incidents<br>Primary reviewer<br>(Table 1)	______	Incident with a risk of harm.<br>Yes/No<br>If Yes Secondary review by physician	______
Harmful incident?<br>Yes/No	______	Classification of incidents<br>Secondary reviewer<br>(Table 1)<br>Use if harmful incident No<br>Stop if no harm identified	______	Type of harm<br>(Table 2)<br>Use if harmful incident Yes<br>Continue with categorization of severity	______
Categorization of severity<br>(Table 3)	______	Assessment of Preventability<br>(Table 4)	______	Number of harmful incidents	______

Classification of incidents, types of harm, severity, and preventability

Table 1. Classification of incidents (no harm identified)	
No incident	No incident in record
Category AB	An incident that may cause error (risk) but did not affect the patient. Example: Inadequate documentation, lack of examinations, vital signs.
Category C	An incident that affected the patient but did not cause any harm. Example: Active treatments/interventions with undesirable results. No harm identified
Category D	An incident that affected the patient and demanded observation or treatment to assure that no harm occurred. Example: Active treatments/interventions with undesirable results where active interventions are required to ensure no harm.

	Table 2. Types of harm (harm identified)
1	Allergic reaction
2	Bleeding, not in connection with surgery or other invasive procedure
3	Bleeding, in connection with surgery or other invasive procedure
4	Harm induced by fall
5	Skin damage or superficial vascular damage
6	Infections incl. thrombophlebitis
7	Harm to organ
8	Failure of vital signs including cardiac arrest
9	Anesthesia-related harm
10	Drug-related harm (non-allergic reaction)
11	Harm caused by medical technology
12	Postpartum/obstetric harm
13	Neurological harm
14	Harm to thorax
15	Harm to extremity
16	Delayed care
17	Other damages, specify

Table 3. Classification of severity (harm identified)	
Category E	Contributed to or resulted in temporary harm that required intervention.
Category F	Contributed to or resulted in temporary harm that required outpatient care, readmission, or prolonged hospital care.
Category G	Contributed to or caused permanent harm.
Category H	An event that required lifesaving intervention within 60 min.
Category I	Contributed to the patient's death.

Table 4. Assessment of preventability (harm identified)	
1	Harm *was not* preventable
2	Harm *was unlikely to be* preventable
3	Harm was likely preventable
4	Harm was preventable
